# Risk of cardiovascular events among celiac disease patients: A meta-analysis review

**DOI:** 10.6026/973206300210375

**Published:** 2025-03-31

**Authors:** Saad Shams, Vanessa Vidaurre Corrales, Ibrahim Marouf Yasin Al Shyyab, Akshat Banga, Ameer Mustafa Farrukh, Moza Ali Alzaabi, Majd Atef Albeetar, Sem Josue Nsanh Yao, Kavya Remala, Mohamad Emad Aldeen Mahfouz, Navneeth Jayaprakash, Sawai Singh Rathore

**Affiliations:** 1 Department of Internal Medicine, University of Oklahoma Health Sciences Center, Oklahoma City, USA; 2 Department of Internal Medicine, Universidad Privada del Valle, Cochabamba, Bolivia; 3 Department of Internal Medicine, University of Sharjah, Sharjah, UAE; 4 Department of Internal Medicine, Mt. Auburn Hospital, Cambridge, USA; 5 Department of Internal Medicine, University of Galway School of Medicine, Galway, Ireland; 6 Department of Internal Medicine, Western Reserve Hospital, Cuyahoga Falls, USA; 7 Department of Internal Medicine, Konaseema Institute of Medical Sciences, Amalapuram, India; 8 Department of Internal Medicine, D.Y. Patil Medical College, Kolhapur, India; 9 Department of Internal Medicine, Dr. Sampurnanand Medical College, Jodhpur, Rajasthan, India

**Keywords:** Celiac disease, cardiovascular events, myocardial infarction, atrial fibrillation, stroke

## Abstract

A review on the association between celiac disease and cardiovascular events using multiple databases and random effects models is of interest. The overall prevalence of cardiovascular events in celiac patients was 9.3%. However, there was no significant increase in overall cardiovascular risk or myocardial infarction compared to the general population. Known data shows that celiac disease was linked to a higher risk of atrial fibrillation, stroke, congestive heart failure and cardiomyopathy. Thus, there is a need to monitor cardiovascular health in celiac patients to mitigate potential risks.

## Background:

Celiac disease is an autoimmune disorder characterized by an abnormal immune response to gluten, a protein found in wheat, barley and rye. It is estimated to impact 1% of the global population and is linked to small intestine inflammation and damage, which can result in a range of gastrointestinal symptoms and mal-absorption of nutrients [1]. The celiac disease is distinguished by a diverse clinical picture and higher morbidity and mortality, owing mostly to its complications, such as sepsis, respiratory infections and malignant states, for example, refractory celiac disease lymphomas and small-bowel carcinoma [2, 3]. Emerging data from the past few years suggests that celiac disease may also raise the risk of cardiovascular events such as stroke, myocardial infarction and atrial fibrillation [4]. Because of the implications for celiac disease patients' care and monitoring, there is a great deal of clinical interest in this possible relationship between cardiovascular disease and celiac disease While some research has indicated that patients with celiac disease have an increased risk of cardiovascular events, other studies have produced contradictory findings [6, 7 and 8]. Therefore, it is interest to report the risk of cardiovascular events among celiac disease patients using a meta-analysis review.

## Methods:

The current systematic review and meta-analysis are processed and communicated in accordance with Preferred Reporting Items for Systematic Reviews and Meta-analyses (PRISMA) guidelines [9].

## Search strategy:

A comprehensive literature search was conducted with PubMed and Embase until March 2024. The MedRxiv and SSNR preprint servers were also filtered. We combined Medical Subject Headings (MeSH) terms and keywords and subsequent search terms were, ([Celiac disease] or [gluten-sensitive enteropathy] or [Celiac Sprue] AND [Cardiovascular events] or [Myocardial Infarction] or [Arrhythmia's] or [Stroke] or [Cardiomyopathy] or [Heart failure]). Investigations came from all over the world and there were no language limitations. The eligibility of the remaining articles was assessed by examining their titles and abstracts after duplicate citations were eliminated. After removing duplicate citations, the eligibility of the remaining articles was evaluated by looking at their titles and abstracts. The PRISMA flow diagram is depicted in Figure 1 - (see PDF).

## Eligibility criteria:

All qualified studies were included in this meta-analysis. To be competent for this meta-analysis, the article must fulfil the subsequent inclusion criteria: (a) an article describing any type of cardiovascular event in Celiac disease; (b) studies with a sample size of ≥ 5 patients. These studies were included irrespective of the patient's age, gender, or ethnicity. The exclusion criteria were predetermined and included the following: (a) case reports, commentaries, reviews, posters and letters to the editor; (b) duplicate publications; and (c) no data regarding the prevalence of cardiovascular events. Following the fulfilment of these requirements, a thorough analysis of the remaining studies and the extraction of data into an Excel table were completed.

## Study selection and quality assessment:

Each author looked over the abstracts and titles of the previously found papers individually. Based on the predetermined eligibility standard, each author identified differences between the studies. Negotiations and an earlier agreement that a third author would assess the differences in opinion helped to resolve the conflict. The quality of the included studies and the risk of bias were evaluated using the Newcastle-Ottawa Scale (NOS) [10]. To evaluate the unique quality of each study, two of us independently used the Newcastle-Ottawa Scale (NOS). Each study assigned a score to the following sections: low bias risk (8-9 points), moderate bias risk (5-7 points) and high bias risk (0-4 points).

## Data extraction:

Authors independently advanced each study's data extraction, which was then cross-checked to reduce errors. From each study, several details were retrieved, including the first author's name, the origin country of the analysis, study design, Sample size, Celiac disease patients, CVD Events, MI events, AF events, Stroke events, Cardiomyopathy events, CHF events, median age and gender (female sex proportion).

## Statistical analysis:

MedCalc® Statistical Software version 19.6.4 (MedCalc Software Ltd, Ostend, Belgium; https://www.medcalc.org; 2021) was utilized for all statistical analyses. The random effect model calculated the pooled prevalence and associated 95% confidence interval (CI). Results for outcome analysis were presented as odds ratios (0Rs) with 95% confidence intervals (CIs) and pooled using the Mantel-Haenszel random-effects model. The I2 statistics were used to assess the heterogeneity of effect size estimates across these studies with I2 (low heterogeneity: I2 ≤ 25%; moderate: 25-50%; high > 75%). Publication bias was explored using funnel plots, Egger's regression test and Begg-Mazumdar's rank correlation test.

## Results and Discussion:

## Characteristics of the included studies:

Preliminary scans in numerous databases yielded 1234 articles. After removing duplicates, 834 were evaluated from this group. After considering the title and abstract, 751 articles were eliminated, leaving 83 papers that could be regarded as plausible for this analysis after passing the article review. Based on a thorough review and inclusion criteria, 22 articles were eventually included in this meta-analysis. Table 1 summarizes the baseline characteristics of the included studies [5, 6, 11, 12, 13, 14, 15, 16, 17, 18, 19, 20, 21, 22, 23, 24, 25, 26, 27, 28, 29- 30]. Figure 2 - (see PDF) depicts the pictorial representation of cardiovascular risk in celiac disease patients. The overall pooled random effects estimate of any cardiovascular event (CVD) in celiac disease patients across multiple studies was 9.3%. This estimate came with a 95% confidence interval (CI) ranging from 4.71% to 15.37%. These statistics were derived from a comprehensive analysis that included data from 13 studies, encompassing a substantial patient population of 371,889 patients diagnosed with celiac disease (Supplementary Figure 1 - see PDF). However, Meta-analysis results also indicated that celiac disease was not associated with an increased risk of any cardiovascular event when compared to a matched cohort from the general population without celiac disease. (OR= 1.27, 95% CI 0.98 to 1.65). This outcome had high associated heterogeneity (I2 = 92%). The result was pooled from 12 studies comprising 364,459 Celiac disease patients (Figure 3 - see PDF).

Similarly, Meta-analysis results indicated that celiac disease was not associated with an increased risk of MI compared to the general population without celiac disease (OR= 1.02, 95% CI 0.74 to 1.41). This outcome had high associated heterogeneity (I2 = 86%). The result was pooled from 6 articles comprising 110,552 Celiac disease patients (Figure 4 - see PDF). Similarly, the overall pooled random effects estimate of myocardial infarction in celiac disease patients across multiple studies was 2%. (95% CI 1.59 to 2.55). The result was pooled from 8 reports comprising 162,438 celiac disease patients (Supplementary Figure 2 - see PDF). Besides, Meta-analysis results indicated that celiac disease was associated with an increased risk of AF compared to the general population without celiac disease (OR= 1.65, 95% CI 1.22 to 2.25). This outcome had high associated heterogeneity (I2 = 86%). The result was pooled from 3 articles comprising 32,837 Celiac disease patients (Figure 5 - see PDF). Similarly, the overall pooled random effects estimate of atrial fibrillation in celiac disease patients across multiple studies was 6.5%. (95% CI 3.78 to 10.07). The result was pooled from 4 reports comprising 40,277 celiac disease patients (Figure 3 - see PDF). Meta-analysis results further indicated that celiac disease was associated with an increased risk of stroke compared to the general population without celiac disease (OR= 1.26, 95% CI 1.16 to 1.36). This outcome had very little associated heterogeneity (I2 = 13%). The result was pooled from 4 articles comprising 47,918 Celiac disease patients (Figure 6 - see PDF). Similarly, the overall pooled random effects estimate of stroke in celiac disease patients across multiple studies was 2.1%. (95% CI 1.64 to 2.71). The result was pooled from 4 reports comprising 47,918 celiac disease patients (Supplementary Figure 4 - see PDF) Celiac disease was also found to be associated with an increased risk of congestive heart failure (OR= 1.18, 95% CI 1.04 to 1.33) and cardiomyopathy (OR= 1.68, 95% CI 1.21 to 2.32) compared to the general population without celiac disease. Both outcomes had no associated heterogeneity (I2 = 0%). However, the results for both outcomes were pooled only from 2 articles with 27,690 and 43,403 Celiac disease patients respectively (Figure 7A - see PDF and Figure 7B - see PDF).

The Newcastle-Ottawa Scale (NOS) was used to assess the risk of bias and quality of incorporated observational studies. Of 22 studies, 8 were high quality and 16 were moderate quality, with an average score of 7.1. Overall, it was determined that the evidence used in these analyses was of moderate quality. Visual inspection of the standard funnel plots for all the analyses was identified as having Moderate symmetry. With the aid of Egger's regression test and Begg-Mazumdar's rank correlation test, the assessment of publication bias was also accompanied. For both these tests, p< 0.05 was considered significant and the analysis was considered to have publication bias. No evident publication bias was witnessed for most analyses done in this study. However, Egger's regression test for CHF and cardiomyopathy outcome had a P value of less than 0.05. Still, the corresponding Begg-Mazumdar rank correlation test showed no evidence of publication bias for this outcome. Hence, this analysis was considered to have very little publication bias (Table 2).

This study aimed to determine the prevalence and risk of cardiovascular events in patients with celiac disease as compared to the general population. The analysis provides significant information on the cardiovascular health of individuals with celiac disease, giving us a better understanding of the potential links between celiac disease and various cardiovascular conditions. The analysis of 13 studies involving 371,889 patients found that the overall pooled estimate of any cardiovascular event (CVD) in patients with celiac disease was 9.3%. Surprisingly, there was no significant increase in the risk of such events in celiac compared to the general population without celiac disease (OR= 1.27, 95% CI 0.98 to 1.65). This observation contrasts with some previous studies that suggested a link between celiac disease and cardiovascular risk [19, 21]. However, the high heterogeneity associated with this finding warrants caution in its interpretation, indicating potential variations in study methodologies or patient populations across the included studies. Likewise, in comparison to the general population, our analysis failed to identify any meaningful correlation between myocardial infarction (MI) risk and celiac disease (OR= 1.02, 95% CI 0.74 to 1.41). This observation challenges the previously published literature, which showed an increased risk of MI in celiac disease [12, 21]. The overall estimate of the prevalence of MI in patients with celiac disease was 2%, indicating a comparatively low incidence of this cardiovascular event in this particular group. Further, we found that Individuals with celiac disease are at an increased risk of developing atrial fibrillation (AF) (OR= 1.65, 95% CI 1.22 to 2.25) and stroke (OR= 1.26, 95% CI 1.16 to 1.36) as compared to the general population. These findings highlight the importance of closely monitoring the cardiovascular health of individuals with celiac disease, especially with regard to arrhythmias and cerebrovascular events. Furthermore, our analysis revealed positive associations between celiac disease and other cardiovascular conditions, such as congestive heart failure (CHF) and cardiomyopathy. Both conditions exhibited increased risk in celiac disease patients compared to the general population. Although these associations were based on a limited number of studies, the absence of heterogeneity strengthens the reliability of these findings. It is consistent with a few previously published studies [31, 32 and 33].

Researchers are still trying to figure out the mechanisms behind cardiovascular events in people with celiac disease. Several possible pathways have been proposed to explain the link between celiac disease and cardiovascular complications. Chronic inflammation and immune dys-regulation, both of which are associated with celiac disease, are one possible mechanism. Individuals with celiac disease may experience systemic inflammation caused by gluten exposure, leading to the production of inflammatory molecules like tumor necrosis factor-alpha (TNF-α), interleukin-1 (IL-1) and IL-6. This systematic inflammation can lead to endothelial dysfunction, oxidative stress and accelerated atherosclerosis, predisposing them to cardiovascular events such as myocardial infarction or stroke [34]. An early sign of atherosclerosis is endothelial dysfunction, which is characterized by impaired vasodilation and the presence of pro-inflammatory and pro-thrombotic endothelial cell traits. Studies show that celiac disease patients exhibit symptoms of endothelial dysfunction, such as reduced flow-mediated dilatation and increased levels of endothelial activation markers like soluble intercellular adhesion molecule-1 (sICAM-1) and von Will brand factor [35, 36]. These findings imply that the pathophysiology of cardiovascular disorders may involve the endothelial dysfunction seen in celiac disease patients. Moreover, celiac disease-related nutrient mal-absorption and deficiencies, especially of vitamins and minerals like magnesium and vitamin D, may increase the risk of cardiovascular disease by affecting blood pressure regulation, cardiac function and arterial stiffness [4, 37]. Moreover, the development of cardiovascular complications like atrial fibrillation and cardiomyopathy may be directly attributed to the autoimmune processes associated with celiac disease, including the generation of autoantibodies that target endothelial cells and cardiac tissue [38]. Furthermore, celiac disease is often accompanied by other comorbidities, such as diabetes mellitus and autoimmune disorders, which themselves are known risk factors for cardiovascular disease. The interplay between these factors may further amplify cardiovascular risk in celiac disease patients [39]. However, further research is needed to fully elucidate the complex interplay between celiac disease and cardiovascular health, including the contribution of genetic predisposition, environmental factors and potential therapeutic interventions targeting inflammation and immune dys-regulation. Understanding these mechanisms is crucial for developing effective strategies to prevent and manage cardiovascular complications in individuals with celiac disease.

Figure 8 - (see PDF) depicts the mechanism of cardiovascular events in Celiac disease. A comprehensive strategy that tackles both gluten-related complications and cardiovascular health is required to prevent cardiovascular risk in people with celiac disease. (Figure 8 - see PDF) Firstly, maintaining a strict gluten-free diet is paramount to managing celiac disease and reducing inflammation, thereby potentially mitigating the risk of cardiovascular events associated with chronic inflammation. Furthermore, it is essential to regularly monitor cardiovascular risk factors like blood pressure, lipid profiles and blood glucose levels. A balanced diet full of fruits, vegetables and whole grains (but not gluten-containing grains) combined with regular exercise can help control weight, improve lipid profiles and improve cardiovascular health in general. Moreover, reducing cardiovascular risk may also result from addressing potential nutritional deficiencies, such as those in vitamin D and B12 that are frequently observed in celiac disease through appropriate supplementation or dietary modifications. In order to provide comprehensive care and customized management strategies for people with celiac disease to effectively reduce their cardiovascular risk, cooperation between healthcare professionals, including gastroenterologists and cardiologists, is crucial [40, 41]. When interpreting the results of the meta-analysis on cardiovascular events in patients with celiac disease, it is important to take into account a few limitations. First off, the pooled estimates and overall interpretation could have been impacted by the heterogeneity among the included studies, which resulted from differences in populations and research methodologies. Furthermore, the reliability and generalizability of the results may be impacted by publication bias as well as the risk of bias in the included studies. Furthermore, there is a chance that bias was introduced into the analysis due to the scarcity of data on particular cardiovascular outcomes. More rigorous study designs and thorough data collection techniques could help address these limitations and improve the validity and applicability of future research in this field.

## Conclusion:

The link between celiac disease and heart disease risk driven by inflammation and nutritional deficiencies is reviewed. A gluten-free diet helps to mitigate risks to a certain extend. Hence, healthcare providers should consider cardiovascular health in celiac patients to improve outcomes.

## Disclosure:

Authors have no potential conflicts of interest to disclose.

## Data availability statement:

The data that support the findings of this study are available from the corresponding author upon reasonable request.

## Funding statement:

No funding sources to declare

## Abbreviations:

CD: Celiac disease

AF: Atrial Fibrillation

CVD: Cardiovascular events

MI: Myocardial Infarction

## Figures and Tables

**Table 1:** Baseline characteristics of included studies

**Study id**	**Year**	**Follow up period**	**Country**	**Study design**	**Sample size**	**Mean age**	**Female percentage**	**NCOS**
Chen *et al.*	2023	11.8 years	UK	Prospective cohort	330,751	55.10±8.14	57.80%	7
Conroy *et al.*	2023	12.4 years (11.5-13.1)	UK	Prospective cohort	469,095	6.9	55.84%	7
Sharma *et al.*	2023	-	USA	Retrospective cohort	2,769,324	-	63.45%	8
Conrad *et al.*	2022	-	UK	Retrospective cohort	22009375	47.5±19.7	61%	6
Gupta *et al.*	2022	-	USA	Retrospective cohort	0	67.5	39.4	7
Naaraayan *et al.*	2021	10 years	USA	Retrospective cohort	1,360,873	58.61±19.68	71.09%	7
Karhus *et al.*	2020	36 years	Denmark	Retrospective cohort	16,776	48.01	56.74%	6
Lebwohl *et al.*	2020	12.5 years	Sweden	Retrospective cohort	296,255	26.53	62.49%	7
Gajulapalli *et al.*	2017	-	USA	Retrospective cohort	48,642,290	52.12	54.30%	8
Heikkilä *et al.*	2017	26 years	Finland	Prospective cohort	6,887	-	54.42%	6
Lebwohl *et al.*	2015	5 years	Sweden	Retrospective cohort	9,725	28.4	64%	7
Emillson *et al.*	2013	1 year	Sweden	Retrospective cohort	0	-	38.08% (out of 2418)	7
Emillson *et al.*	2012	10.4±6.4	Sweden	Retrospective cohort	173,500	10.3	61.92%	8
Hervonen *et al.*	2012	5 years	Finland	Prospective cohort	1074	-	43.02%	7
Ludvigsson *et al.*	2012	9 years	Sweden	Retrospective cohort	170,493	-	62.25%	8
Emilsson *et al.*	2011	9 years	Sweden	Retrospective cohort	170,368	-	62.35%	6
Ludvigsson *et al.*	2011	8 years	Sweden	Retrospective cohort	0	-	61.07%	8
Wei *et al.*	2008	3.7 years	UK	Retrospective cohort	5904	46±18.3	65.54%	7
Ludvigsson *et al.*	2007	>1 year	Sweden	Retrospective cohort	77476	N/A	59.47%	8
Elfström *et al.*	2007	-	Sweden	Retrospective cohort	84183	-	58.94%	7
West *et al.*	2004	-	UK	prospective cohort	0	-	67.90%	7
Peters *et al.*	2003	8.1 years	Sweden	Retrospective cohort	10,032	17.4	58.43%	8

**Table 2:** Publication bias tests

**Outcome**	**Egger's regression test**	**Begg-Mazumdar's rank test**
Any Cardiovascular event	P = 0.6887	P = 0.4929
Myocardial Infarction Outcome	P = 0.3208	P = 0.3476
Atrial Fibrillation Outcome	P = 0.9316	P = 0.6015
Stroke Outcome	P = 0.1495	P = 1.0000
Congestive heart failure Outcome	P < 0.0001	P = 0.3173
Cardiomyopathy Outcome	P < 0.0001	= 0.3173
